# Lumbar Spine Instability Fracture in a Patient With Residual Adolescent Idiopathic Scoliosis: A Case Report

**DOI:** 10.7759/cureus.86210

**Published:** 2025-06-17

**Authors:** Yuta Masaki, Tomohito Mukaihata, Yasuhiro Shiga, Ko Takano, Seiji Ohtori

**Affiliations:** 1 Department of Orthopaedic Surgery, Graduate School of Medicine, Chiba University, Chiba, JPN; 2 Spine Center, Katori Omigawa Medical Center, Katori, JPN; 3 Department of Orthopaedic Surgery, New Tokyo Hospital, Matsudo, JPN

**Keywords:** adolescent idiopathic scoliosis, lumbar burst fractures, lumbar spine instability, posterior thoracolumbar fusion, residual scoliosis

## Abstract

Lumbar spine instability fractures in patients with residual adolescent idiopathic scoliosis (AIS) are rare, and optimal treatment strategies remain unclear. Surgical management must consider both immediate fracture stabilization and the potential need for future scoliosis correction.

A 24-year-old woman with residual AIS (Cobb angles: 47° thoracic, 60° lumbar) sustained an AO Spine Classification Type B2 lumbar fracture (L2) after falling from the second floor. She presented with severe back pain but no neurological deficits. Radiographic evaluation confirmed vertebral body collapse and posterior ligamentous injury. Posterior fixation alone was chosen over combined anterior-posterior fixation to maintain future options for scoliosis correction. The patient recovered uneventfully, achieving solid bone fusion by seven months postoperatively, at which time the implants were also removed. At the two-year follow-up, she remained asymptomatic with no progression of scoliosis or instability.

For lumbar fractures in patients with residual AIS, surgical decision-making should consider future scoliosis management. Posterior fixation alone may be a viable strategy to ensure both stability and surgical flexibility.

## Introduction

Lumbar spine instability fractures in patients with residual adolescent idiopathic scoliosis (AIS) are rare [1,2]. AIS often stabilizes after growth, but some patients retain significant curvature into adulthood. This residual deformity can alter spinal mechanics and may complicate trauma cases.

AO Spine Classification Type B2 fractures involve posterior tension band injuries and often require surgery, especially when instability is present. In this case, we report a traumatic AO Type B2 fracture at the apex of a lumbar curve in a patient with residual AIS. Posterior fixation alone was selected to maintain future surgical options for scoliosis correction.

## Case presentation

**Figure 1:**
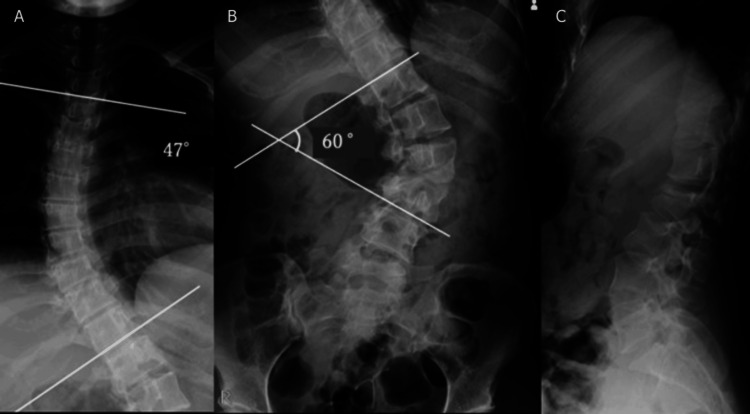
Preoperative radiographs showing severe scoliosis The thoracic spine shows a Cobb angle of 47° (A), and the lumbar spine has a Cobb angle of 60° at the time of injury (B).

A 24-year-old woman with schizophrenia and mild intellectual disability was diagnosed with AIS (Cobb angles: 51° thoracic, 70° lumbar) at age 12. She was asymptomatic, under observation, but dropped out of follow-up after one year. Before the injury, she was independent in her activities of daily living (ADLs) and lived in a group home. She jumped from the second floor after a distressing incident at her group home, landing on her buttocks and sustaining the injury. The day after the injury, she began experiencing back pain and difficulty moving. She called for emergency medical assistance and was transported to her previous doctor, who referred her to our hospital for further treatment. Physical examination revealed low back pain but no paralysis of the extremities, sensory disturbances, or cerebrospinal disturbances. Radiographs showed L2 vertebral collapse with severe scoliosis (Cobb angles: 47° thoracic, 60° lumbar) (Figure 1).

**Figure 2:**
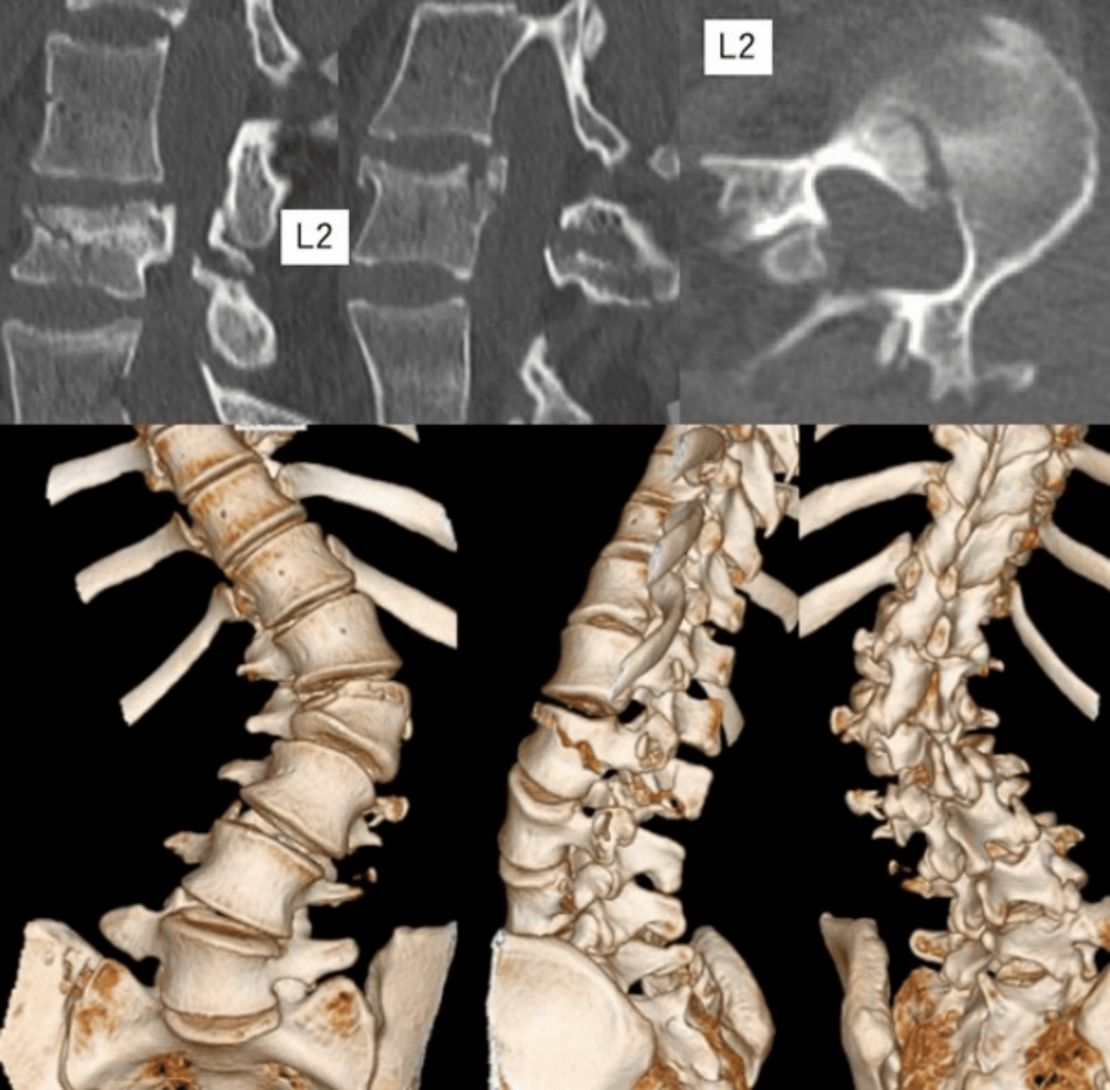
Preoperative computed tomography image of the lumbar spine The fracture line of the second lumbar vertebra extends from the anterior to the posterior element, indicating an AO Spine Classification Type B2 fracture.

Computed tomography (CT) revealed a fracture line extending from the anterior element to the posterior element, and magnetic resonance imaging (MRI) indicated damage to the posterior ligament complex (Figure 2). An AO Type B2 fracture was diagnosed, warranting surgical intervention.

**Figure 3:**
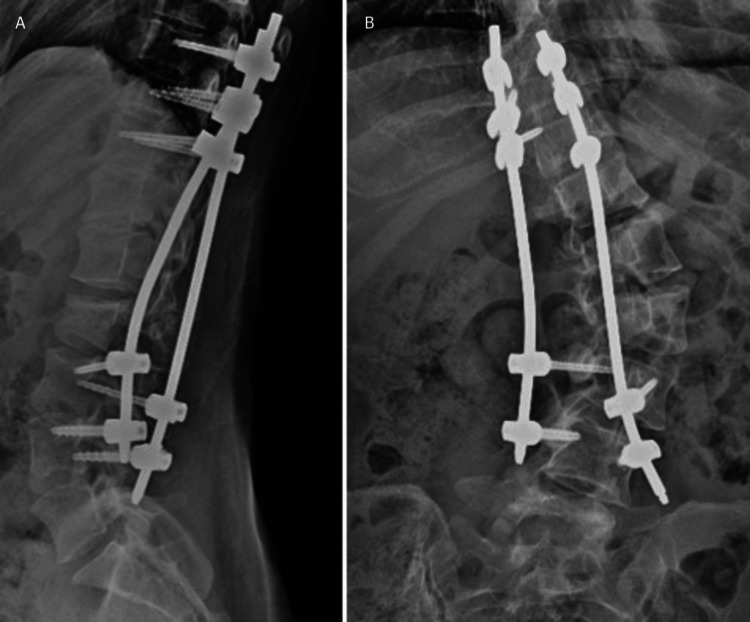
Postoperative radiographs showing posterior thoracolumbar fusion using pedicle screws Screws were placed in the ninth to 11th thoracic vertebrae (A) and the third to fourth lumbar vertebrae (B), secured with rods.

Given the severe vertebral body compression, anterior-posterior fusion was considered, but posterior fusion alone was chosen to preserve options for future anterior scoliosis correction. On the 11th day post-injury, posterior thoracolumbar fusion was performed using CT navigation. Screws were inserted into the ninth to 11th thoracic vertebrae and the third to fourth lumbar vertebrae, and these were secured with rods (Figure 3).

**Figure 4:**
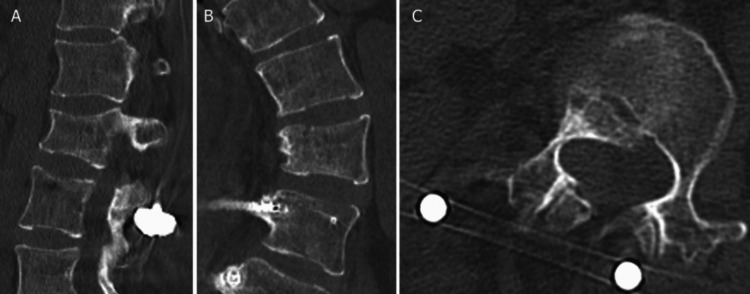
Seven-month postoperative computed tomography images of the lumbar spine (A-C) The radiographs showing successful bone fusion without screw loosening or misalignment.

A rigid corset was created and worn postoperatively. Head-of-bed elevation was limited to 30° until four weeks postoperatively, after which rehabilitation continued without restriction based on pain levels. The patient’s back pain resolved, and she was discharged two months post-surgery. Seven months after the surgery, bone fusion was confirmed, and nail extraction was performed (Figure 4).

**Figure 5:**
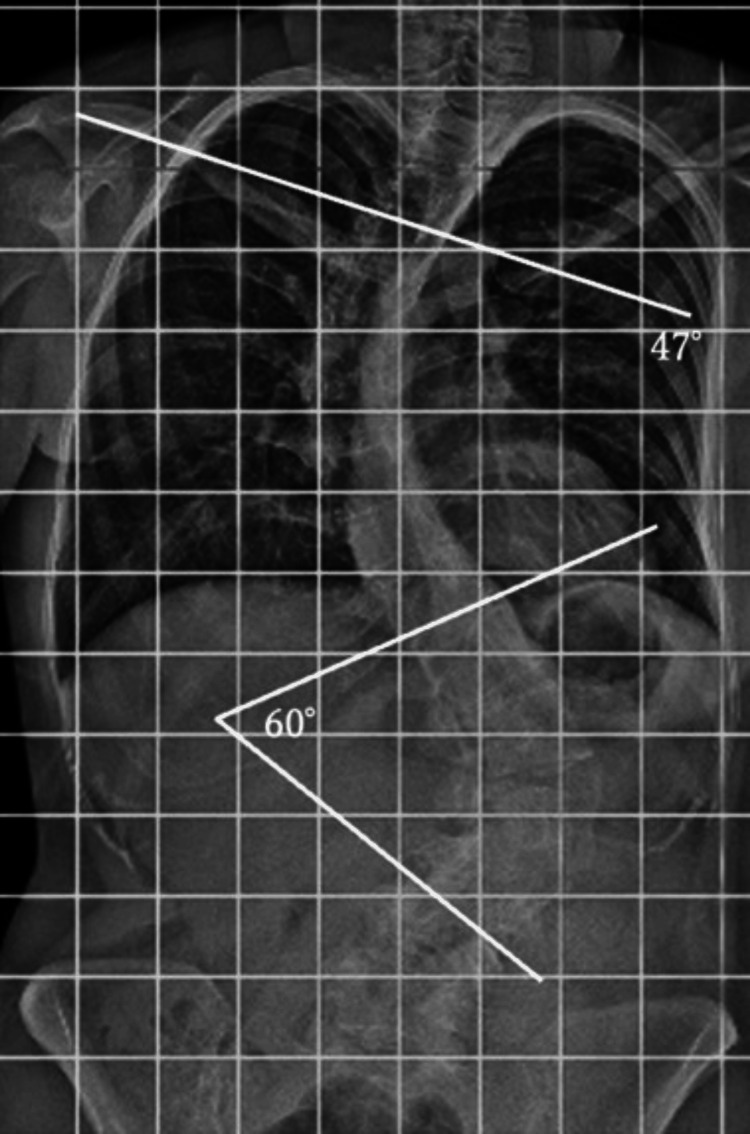
Two-year postoperative whole spine X-ray The radiograph shows no progression of scoliosis.

Two years have passed since the initial surgery, with no reported pain, no disturbance in ADLs, and no worsening of spinal alignment (Figure 5).

## Discussion

To our knowledge, no prior reports have described vertebral fractures specifically in patients with residual AIS [1]. Treatment of AO Type B2 fractures with good bone quality typically involves fixation one level above and below the fracture. However, anterior fixation is often necessary in severe vertebral body compression [3,4]. Observing adult scoliosis, including residual AIS, is the standard approach in asymptomatic young patients with a Cobb angle of ≤45°. Surgery is considered when scoliosis progresses or symptoms such as pain arise, often owing to disc degeneration or bone fragility associated with aging or menopause. Although posterior corrective fusion is the most common technique, anterior corrective fusion may be indicated for large correction angles or to shorten the fixation range [5,6]. In this case, the Cobb angles were 47° and 60° in the thoracic and lumbar spines, respectively, indicating significant scoliosis. Anterior fixation and vertebral body replacement could hinder future scoliosis correction, such as vertebral osteotomy, due to adhesions and implant constraints. This could hinder scoliosis correction. Given these considerations, the decision regarding the fixation strategy was challenging. After discussions with the patient and her family, posterior fixation without an anterior procedure was chosen to preserve options for future corrective surgery. As anterior fixation was not performed, extended bed rest was prescribed to prevent screw loosening or loss of correction. However, considering the absence of scoliosis-related pain prior to the injury and the invasiveness of scoliosis correction surgery involving both thoracic and lumbar regions, it was decided to treat only the fracture.

## Conclusions

A 24-year-old patient with residual AIS and an L2 fracture was successfully treated with posterior fixation alone, avoiding anterior instrumentation. This approach was chosen to preserve the possibility of future scoliosis correction, considering the patient’s significant residual lumbar curve.

In cases of vertebral fractures in patients with residual AIS, surgical strategies should take into account not only fracture stability but also the potential need for future deformity correction. Posterior fixation alone can be a viable option when long-term spinal alignment planning is necessary.
